# Synergistic Impact of Body Mass Index and Cognitive Function on All-Cause Mortality in Older Adults: A Nationwide Longitudinal Study

**DOI:** 10.3389/fendo.2021.620261

**Published:** 2021-06-29

**Authors:** Ke Han, Wangping Jia, Shengshu Wang, Wenzhe Cao, Yang Song, Jianwei Wang, Miao Liu, Shanshan Yang, Yao He

**Affiliations:** ^1^ Medical School of Chinese PLA, Beijing, China; ^2^ Institute of Geriatrics, Beijing Key Laboratory of Aging and Geriatrics, National Clinical Research Center for Geriatrics Diseases, State Key Laboratory of Kidney Disease, The 2nd Medical Center of Chinese PLA General Hospital, Beijing, China; ^3^ Department of Statistics and Epidemiology, Medical School of Chinese PLA, Beijing, China; ^4^ Department of Disease Prevention and Control, The 1st Medical Center, Chinese PLA General Hospital, Beijing, China

**Keywords:** cognition, body mass index, malnutrition, older adults, mortality

## Abstract

**Background:**

Body mass index (BMI) and cognitive function are independent predictors of mortality risk. However, little is known about the combined impact of BMI and cognitive function on the risk of all-cause mortality in older adults. In this study, we aimed to examine the associations between BMI, cognitive function, and all-cause mortality, including between-factor interactions, in the general population of older adults in China.

**Methods:**

We used the data between 2011 and 2018 from the Chinese Longitudinal Healthy Longevity Survey that included adults aged ≥65 years residing in the 23 provinces of China. The association between BMI and cognitive function on all-cause mortality was examined with the Cox proportional hazards regression model.

**Results:**

The study included 8,293 Chinese older adults. Low BMI (underweight) and cognitive impairment were associated with the highest risk of death after adjustments [hazard ratio (HR) = 2.18; 95% confidence interval (CI), 1.96–2.41]; this combined effect was more prominent among adults aged <100 years and women. In addition, there was an interaction effect of BMI and cognitive impairment on all-cause mortality (P <0.001). Concurrently, among older adults with normal cognition, the risk of mortality related to underweight was higher than among their cognitively impaired counterparts [55% (normal cognition) *vs.* 38% (cognitive impairment)].

**Conclusions:**

Low BMI (underweight) and cognitive impairment were independently and jointly associated with increased risk of all-cause mortality among Chinese older adults, and females showed a stronger effect in this association. The association between BMI and mortality was more pronounced in the participants with normal cognition than in their cognitively impaired counterparts.

## Introduction

Body mass index (BMI) has been previously investigated in several studies dedicated to older adults and is considered a predictor of premature mortality in this population. BMI has been reported to follow a U-shaped (1, 2) or J-shaped (3–5) association with mortality, and the impact of obesity on mortality risk may decrease with age (6) and over time (7). However, accruing evidence demonstrates that for community-dwelling older adults, low BMI may have a more pronounced impact on mortality risk than high BMI (8, 9). Compared to Western populations, the Chinese population has lower BMI and a higher proportion of underweight individuals (10), suggesting that the impact of low BMI on outcomes, including mortality, in the Chinese older adults may be distinct and warrants further investigation.

Cognitive impairment (CI) is a major public health concern because it is associated with adverse economic and socio-psychological outcomes (11). In aging societies, the prevalence of CI is gradually increasing, in particular, among older adults (12), and the number of people affected is expected to exceed 140 million by 2050 (13). China has one of the fastest aging populations worldwide (14), which translates to a high economic burden associated with CI (15). CI contributes toward a loss of capacity for self-care, reduced quality of life, and increased risk of disability and premature mortality (16, 17).

There is a close association between BMI and cognitive function in older adults. Previous studies of older adults across various ethnic groups revealed that being underweight is associated with an increased risk of cognitive decline (18–20) and that BMI and cognitive function share a genetic basis (21, 22). Although these factors are important contributors to the survival and well-being of older adults, their combined effects on the risk of mortality remain unclear and require further investigation. Hence, in this study, we aimed to examine the independent and synergistic impact of BMI and CI on the risk of all-cause mortality using data from the Chinese Longitudinal Healthy Longevity Survey (CLHLS), conducted on a nationally representative sample of older adults in China.

## Methods

### Study Setting and Participants

We used the 2011-2018 longitudinal data of the CLHLS, a nationwide, prospective, longitudinal study of Chinese community-dwelling older adults. The CLHLS used a multistage, stratified cluster sampling, and recruited older Chinese adults from half the cities or counties in 23 out of the 31 provinces of mainland China, constituting 85% of the Chinese population. A detailed description of sampling frame and investigation methods of CLHLS has been outlined in previous reports (23).

Participants in this study were recruited from the sixth wave (2011/2012) of CLHLS. Among the 9765 participants enrolled in the baseline interview, we included all older adults aged 65 or above (excluding 86 participants with age mismatch) and with BMI information and cognitive assessment (excluding 530 participants with missing data) at baseline. After exclusion of 856 subjects lost to follow-up, a total of 8293 older adults were included for the analysis. **Figure S1** shows the full process of the inclusion and exclusion of the participants.

Ethical approval was granted by the Ethics Committee of Peking University and Duke University. Written informed consent was obtained from all individual participants included in the study.

### Data Collection

#### BMI

Weight and height were measured during the survey. Body mass index was calculated as bodyweight (kg) divided by squared body height (m^2^). According to the recommended classifications for Chinese adults (24), we define underweight as BMI <18.5 kg/m^2^, normal as 18.5-23.9 kg/m^2^, overweight as 24-27.9 kg/m^2^, and obesity as ≥ 28 kg/m^2^.

#### Cognitive Function

Cognitive performance was assessed by the Chinese version of Mini-Mental State Examination (MMSE), which is adapted from the scale developed by Folstein and colleagues (25). It measures cognitive function domains including orientation, recognition, attention, memory and language. All questions were answered by the respondent in person during the face-to-face interview. About 2% of the interviewees were “unable to answer” some of the questions due to low cognitive function as the interviewer indicated (26). Therefore, we encoded these answers as incorrect answers (scored 0) during data processing. The total score ranges from 0 to 30, with higher scores indicating better cognitive functions (27). Cognitive impairment (CI) was defined using education-based cutoff points (28, 29): < 18, participants with no formal education; < 24, participants with 1-6 years of education; < 25, participants with more than 6 years of education; those who did not to meet these criteria were defined as having normal cognition.

#### All-Cause Mortality

For the respondents who died before the next wave of surveys, a specific questionnaire was used to obtain comprehensive death information. Survival status and date of death were collected from official death certificates when available. Otherwise, the information was collected from close relatives who were familiar with the deceased or the local residents committee. Survival time was calculated from the date of the baseline interview to the date of death (for participants who died) or the interview date of the last follow-up survey (for participants who were alive). Those who cannot be contacted were assigned a “lost follow-up” status and were censored in the analysis.

#### Covariates

Covariate information were collected from the structured questionnaire. Demographic information included age (continuous), sex (men or women), ethnicity (Han or others), residence (city, town or rural), and education (≥ 1 year of schooling or < 1 year of schooling). We also considered lifestyle characteristics such as smoking status (current, former or never), alcohol drinking status (current, former or never), and weekly exercise (current, former or never) and self-reported medical history including hypertension, diabetes mellitus, cardiovascular disease, stroke and cerebrovascular disease, respiratory disease and cancer. Impairment in activities of Daily Living Disorder (ADL) was defined as a participant being dependent in at least one aspect of using the toilet, bathing, indoor activities, dressing, eating, or continence. Whether the older adults suffered from spinal deformity was judged by the interviewer based on observation rather than direct inquiry in order to avoid offending the participants. In addition, quality of sleep (five levels from “very good” to “very bad”) and access to adequate medical service (yes *vs.* no) were collected.

### Statistical Analyses

In all analyses, we categorized the BMI and cognition into binary variables and created a 4-level joint variable including: BMI≥18.5 kg/m^2^ and normal cognition, BMI<18.5 kg/m^2^ and normal cognition, BMI≥18.5 kg/m^2^ and CI, and BMI<18.5 kg/m^2^ and CI.

Baseline characteristic of all participants were represented by the 4 groups. Continuous variables were expressed as mean and standard deviation (SD) and categorized variables were expressed as number and percentage. The differences between the means and proportions of the two groups were compared by analysis of variance (ANOVA) and chi-square test. We evaluated all-cause mortality by the 4-level joint variable of BMI/CI with Kaplan-Meier survival plots. Equality of survival rates was tested with log-rank tests.

We conducted multivariable Cox proportional hazard models to calculate the hazard ratios (HRs) for all-cause mortality by the four groups. The basic model was adjusted for age, sex, ethnicity, residence, education; and the fully adjusted model additionally adjusted for smoking, alcohol drinking, weekly exercise, ADL impairment, spinal deformity, hypertension, diabetes mellitus, cardiovascular disease, stroke and cerebrovascular disease, respiratory disease and cancer. The significance of multiplicative interaction between BMI levels and CI for all-cause mortality was tested by adding cross-product terms in the models. Based on the fully adjusted Cox model, we used restricted cubic splines with knots at 18.5 kg/m^2^ (BMI) and 24 points (MMSE) (30) to flexibly model the association of BMI and cognition performance with all-cause mortality. In this part, we also observed the association between BMI and all-cause mortality by CI categories.

In order to further observe the influence of important confounding factors such as age and sex in this association, we conducted subgroup analyses based on the fully adjusted Cox model above. We also tested the interactions between the 4-level joint variable of BMI/CI and subgroup factors for all-cause mortality.

We performed several sensitivity analyses to evaluated the robustness of our results: 1) additionally adjusting for medical treatment accessibility as the deprivation of medical treatment may increase the mortality risk (31); 2) additionally adjusting for sleep quality which is an important factor related to BMI (32), CI (33) and all-cause mortality (34); 3) excluding the participants with spinal deformity; 4) excluding the participants whose BMI > 24 kg/m^2^; and 5) excluding the participants who died in the first year. All statistical analyses were performed using R (version 3.6.3) and Empower Stats (X&Y Solutions, Inc., Boston, MA). A p-value < 0.05 (2-tailed) was considered statistically significant.

## Results

**Figure 1:**
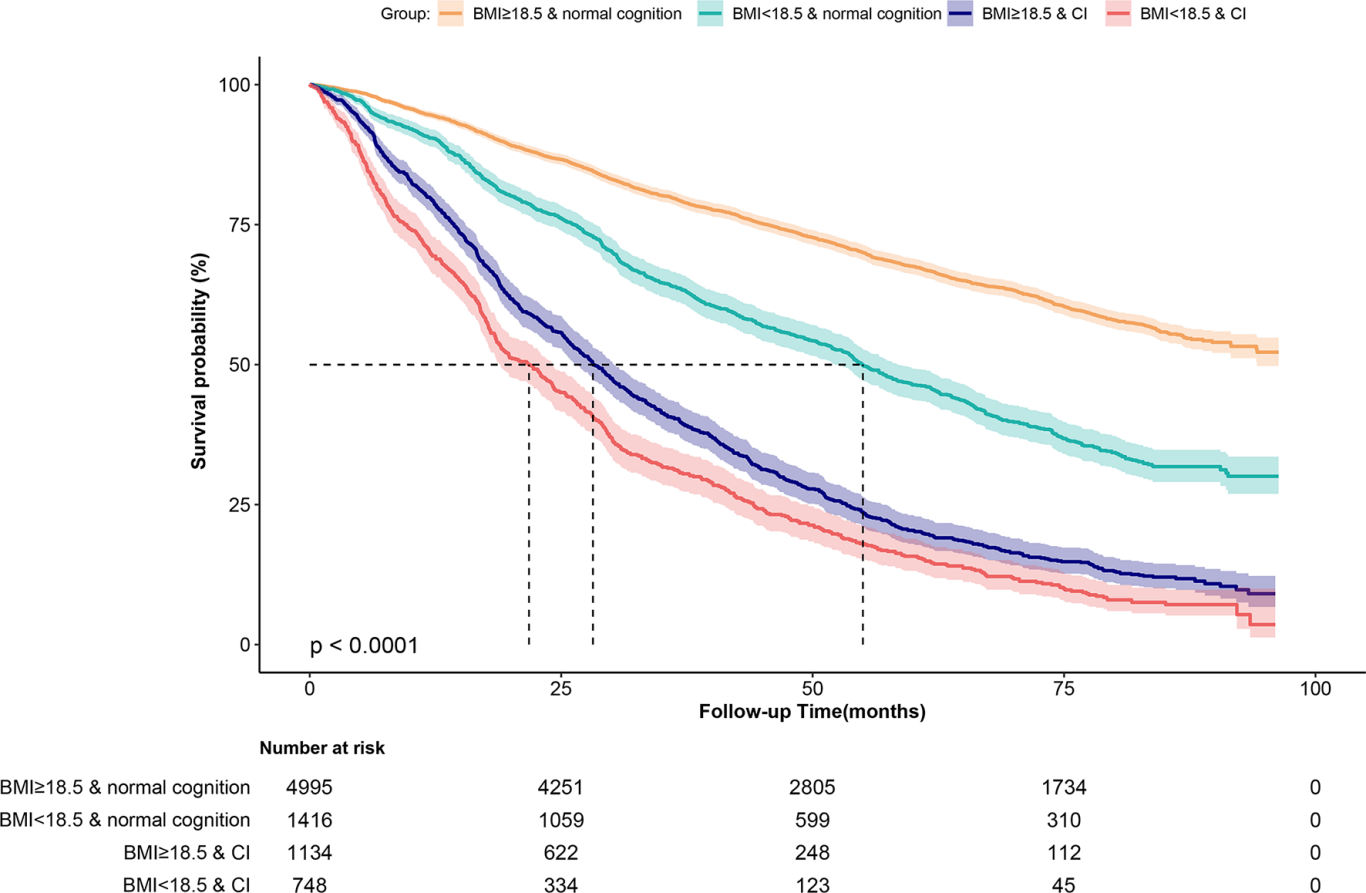
Kaplan-Meier survival curves for Chinese older adults stratified by the 4-level joint BMI/cognition groups.

**Table 1** presents the baseline characteristics of 8,293 (4,555 women) participants included in this study, by the joint variable of BMI and CI. Participants’ mean age was 85.53 (SD, 11.07) years, ranging between 65 and 104 years. The average BMI was 21.20 (SD, 4.23) kg/m^2^ and the average MMSE score was 22.77 (SD, 8.82). Among all participants, 2,164 (764 men and 1,400 women) older adults were underweight, accounting for 26.09% of the sample. In addition, 1,882 (553 men and 1,329 women) older adults had CI, accounting for 22.70% of the sample. Participants who had both CI and low BMI (underweight) were more likely to be older, female, having a lower level of education, having lower ADL scores, and having no history of smoking or alcohol use, or a weekly exercise habit than their counterparts. There were differences in the prevalence of spinal deformity, hypertension, diabetes mellitus, cardiovascular diseases, stroke and cerebrovascular diseases, respiratory diseases and dementia between the groups. The Kaplan-Meier survival curves revealed distinct outcome trajectories for the groups (log-rank p-value <0.001, **Figure 1**). The median survival time was 54.93, 28.16, and 21.75 months for the groups with normal cognition/underweight, CI/normal weight/obesity, and CI/underweight, respectively.

**Table 1:** Baseline characteristics of participants by BMI and cognition categories.

Characteristics	Overall (n=8293)	BMI≥18.5 & normal cognition (n=4995)	BMI<18.5 & normal cognition (n=1416)	BMI≥18.5 & CI (n=1134)	BMI<18.5 & CI (n=748)	P-value
Age (year)	85.53 ± 11.07	81.78 ± 10.04	86.90 ± 10.07	93.49 ± 9.04	95.85 ± 8.44	<0.001
BMI (kg/m^2^)	21.20 ± 4.23	22.93 ± 3.65	16.81 ± 1.52	22.28 ± 3.73	16.38 ± 1.59	<0.001
MMSE score	22.77 ± 8.82	27.15 ± 3.00	26.19 ± 3.39	8.92 ± 7.01	7.99 ± 6.87	<0.001
Sex						<0.001
Men	3738 (45.07)	2583 (51.71)	602 (42.51)	391 (34.48)	162 (21.66)	
Women	4555 (54.93)	2412 (48.29)	814 (57.49)	743 (65.52)	586 (78.34)	
Ethnicity						0.038
Han	7845 (94.60)	4752 (95.14)	1326 (93.64)	1070 (94.36)	697 (93.18)	
Others	448 (5.40)	243 (4.86)	90 (6.36)	64 (5.64)	51 (6.82)	
Residence						<0.001
City	1360 (16.40)	905 (18.12)	162 (11.44)	184 (16.23)	109 (14.57)	
Town	2596 (31.30)	1623 (32.49)	425 (30.01)	338 (29.81)	210 (28.07)	
Rural	4337 (52.30)	2467 (49.39)	829 (58.55)	612 (53.97)	429 (57.35)	
Years of education						<0.001
<1	4865 (58.66)	2537 (50.79)	887 (62.64)	846 (74.60)	595 (79.55)	
≥1	3428 (41.34)	2458 (49.21)	529 (37.36)	288 (25.40)	153 (20.45)	
Smoking						<0.001
Current	1528 (18.43)	1011 (20.24)	305 (21.54)	131 (11.55)	81 (10.83)	
Former	1327 (16.00)	870 (17.42)	219 (15.47)	158 (13.93)	80 (10.70)	
Never	5438 (65.57)	3114 (62.34)	892 (62.99)	845 (74.51)	587 (78.48)	
Alcohol drinking						<0.001
Current	1449 (17.47)	968 (19.38)	251 (17.73)	147 (12.96)	83 (11.10)	
Former	1218 (14.69)	779 (15.60)	184 (12.99)	174 (15.34)	81 (10.83)	
Never	5626 (67.84)	3248 (65.03)	981 (69.28)	813 (71.69)	584 (78.07)	
Weekly exercise						<0.001
Current	2818 (33.98)	2058 (41.20)	444 (31.36)	192 (16.93)	124 (16.58)	
Former	990 (11.94)	495 (9.91)	185 (13.06)	204 (17.99)	106 (14.17)	
Never	4485 (54.08)	2442 (48.89)	787 (55.58)	738 (65.08)	518 (69.25)	
ADL impairment	2028 (24.46)	693 (13.88)	270 (19.07)	606 (53.44)	459 (61.36)	<0.001
Spinal deformity	3144 (37.91)	1474 (29.51)	576 (40.68)	678 (59.79)	416 (55.61)	<0.001
Hypertension	4718 (56.89)	3025 (60.56)	696 (49.15)	635 (56.00)	362 (48.40)	<0.001
Diabetes	341 (4.11)	279 (5.59)	26 (1.84)	28 (2.47)	8 (1.07)	<0.001
Cardiovascular disease	1011 (12.19)	690 (13.81)	150 (10.59)	112 (9.88)	59 (7.89)	<0.001
Stroke and cerebrovascular disease	642 (7.74)	393 (7.87)	72 (5.08)	127 (11.20)	50 (6.68)	<0.001
Respiratory disease	979 (11.81)	587 (11.75)	206 (14.55)	117 (10.32)	69 (9.22)	<0.001
Cancer	65 (0.78)	41 (0.82)	13 (0.92)	9 (0.79)	2 (0.27)	0.394
Parkinson’s disease	68 (0.82)	38 (0.76)	6 (0.42)	14 (1.23)	10 (1.34)	0.05
Dementia	235 (2.83)	42 (0.84)	8 (0.56)	112 (9.88)	73 (9.76)	<0.001

Data were reported as the mean ± standard deviation (SD) for continuous variables and number (%) for categorized variables.

Differences between groups were evaluated by analysis of variance (ANOVA) or chi-square test.

BMI, body mass index; CI, cognitive impairment; ADL, activity of daily living.

During the 7-year follow-up period, 856 participants were lost to follow-up (drop-out rate, 9.36%). The participants lost to follow-up were older and less likely to regularly consume alcohol or have a spinal deformity, had lower MMSE scores, town-dwelling, had lower levels of education, and a higher prevalence of cerebrovascular diseases and stroke (see **Table S1**) than their retained counterparts.

### BMI and Cognitive Function, and All-Cause Mortality

**Figure 2:**
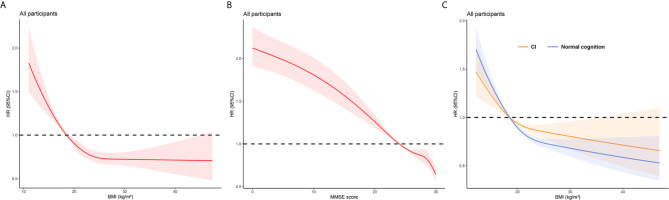
Restricted cubic splines for the association of BMI and MMSE score with all-cause mortality. **(A)** Association of BMI and all-cause mortality in all participants. **(B)** Association of MMSE score and all-cause mortality in all participants. **(C)** Association of BMI and all-cause mortality by cognitive functions in all participants. All models were adjusted for age, sex, ethnicity, residence, education, smoking, alcohol drinking, weekly exercise, ADL impairment, spinal deformity, hypertension, diabetes mellitus, cardiovascular disease, stroke and cerebrovascular disease, respiratory disease and cancer.

In total, 4,212 (1,836 men and 2,376 women) participants died during an unweighted median follow-up period of 40.44 (interquartile range, 25.53–79.24) months. Restricted cubic splines flexibly modeled and visualized the independent associations between BMI and cognitive function, and all-cause mortality (**Figures 2A, B**). There was a non-linear association between BMI and the MMSE score, and all-cause mortality in all participants (all P for non-linear <0.001). Low BMI was consistently associated with increased risk of all-cause mortality, but further increase of BMI values had a marginal impact on the risk of all-cause mortality. Compared to the reference point, as the MMSE score increases from 0 to 30, the risk of all-cause mortality continues to decrease.

**Table 2** presents the combined association between BMI and CI, and all-cause mortality. The normal cognition and BMI ≥18.5 kg/m^2^ group was used as the reference. In the unadjusted model, CI [HR=1.94, 95% confidence interval (CI), 1.79–2.11], low BMI (underweight) (HR=3.88, 95% CI, 3.58-4.20), and the combination of these factors (HR=5.00, 95% CI, 4.57–5.48) were associated with a higher risk of all-cause mortality. In the adjusted model, compared with the reference group, CI, low BMI (underweight), and the combination of these factors yielded the following effect estimates: HR=1.55 (95% CI, 1.42–1.68), 1.80 (95% CI, 1.64–1.96), and 2.18 (95% CI, 1.96–2.41), respectively.

**Table 2:** Hazard ratios for the combined associations of BMI and cognitive impairment with all-cause mortality.

	No. of deaths	HRs for all-cause mortality		
		Unadjusted model	Basic model^a^	Fully adjusted model^b^
Normal cognition				
BMI≥18.5 kg/m^2^	1825	Ref.	Ref.	Ref.
BMI<18.5 kg/m^2^	827	1.94 (1.79, 2.11)	1.52 (1.39, 1.65)	1.55 (1.42, 1.68)
CI				
BMI≥18.5 kg/m^2^	917	3.88 (3.58, 4.20)	2.10 (1.93, 2.29)	1.80 (1.64, 1.96)
BMI<18.5 kg/m^2^	643	5.00 (4.57, 5.48)	2.48 (2.25, 2.74)	2.18 (1.96, 2.41)
P for interaction	–	<0.001	<0.001	<0.001

HR, hazard ratio; BMI, body mass index; CI, cognitive impairment.

^a^Basic model: adjusted for age, sex, ethnicity, residence, education.

^b^Fully adjusted model: additionally adjusted for smoking, alcohol drinking, weekly exercise, ADL impairment, spinal deformity, hypertension, diabetes mellitus, cardiovascular disease, stroke and cerebrovascular disease, respiratory disease and cancer.

There was a significant interaction effect of BMI and CI on all-cause mortality in all models (p-values for interaction < 0.001). Compared to other groups, the combination of lower BMI (underweight) and CI was associated with the highest risk of all-cause mortality in the fully adjusted model. The impact of BMI on the risk of mortality was the higher among people with normal cognition than that among cognitively impaired participants [55% (normal cognition) *vs.* 38% (CI)]. In an analysis stratified by cognitive function categories, restricted cubic splines for remained a reverse-J shape and we found that the all-cause mortality risk varied flatter in participants with normal cognition (**Figure 2C**). The difference was more significant in women and participants aged <100 years than in men or centenarians (**Figure S2**).

### Subgroup and Sensitivity Analyses

**Figure 3:**
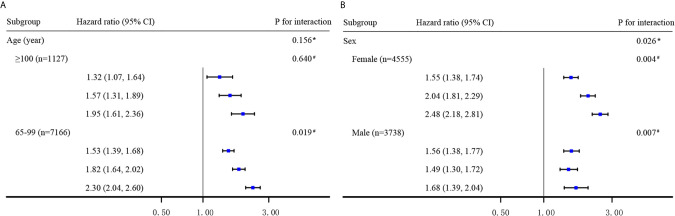
Hazard ratios for the combined associations of BMI and CI with all-cause mortality according to the classification of age **(A)** and sex **(B)**. All models were adjusted for age, sex (not in Panel **B**), ethnicity, residence, education, smoking, alcohol drinking, weekly exercise, ADL impairment, spinal deformity, hypertension, diabetes mellitus, cardiovascular disease, stroke and cerebrovascular disease, respiratory disease and cancer. The estimated HRs for each subgroup are compared with the BMI≥18.5 kg/m^2^ and normal cognition group (not shown) and presented in the order of BMI<18.5 kg/m^2^ and normal cognition group, BMI≥18.5 kg/m^2^and CI group, and BMI<18.5 kg/m^2^ and CI group, respectively. ^*^ Interaction between the 4-level joint variable of BMI/CI and age or sex subgroup on all-cause mortality. ^#^ Interaction between BMI levels and cognitive impairment on all-cause mortality.

Subgroup analyses revealed that the combined impact of BMI and CI on mortality risk was more prominent among adults aged <100 years and women than among centenarians or men (**Figure 3**). We found the associations between the joint variable of BMI/CI and mortality by age and sex showed significant interaction effects (P=0.156 and 0.026, respectively). In the group aged >100 years, the differences between estimated effect sizes associated with different BMI categories [32% (normal cognition) vs. 38% (CI)] were inconsistent with the estimates of the same parameters for the overall sample; however, the interaction effect of BMI and CI on all-cause mortality in centenarians was not significant (P for interaction = 0.640). In particular, we found in the group younger than 80 years that when underweight, CI existed alone or both existed, the association with the risk of all-cause mortality was the strongest in all age groups, but no significant interaction effect was found in this group (P for interaction = 0.300, **Table S2**).

After additional adjustments for access to medical services and quality of sleep, the combined impact of BMI and CI on all-cause mortality remained similar to that estimated in the primary analysis (**Table S3**). However, the combined impact of BMI and CI on all-cause mortality became more pronounced after participants with spinal deformities were excluded. Exclusion of the participants who were overweight or obese (BMI >24 kg/m^2^) at baseline (n = 1,728) did not affect the effect estimates. Exclusion of the participants who died in the first year (n = 860) slightly decreased the magnitude of the effect estimates. All models in sensitivity analyses demonstrated the stability of interaction effects between binary BMI categories and CI on the risk of all-cause mortality.

## Discussion

In this large prospective community-based study of 8,293 Chinese older adults, we observed a reverse J-shaped association between BMI and all-cause mortality and a positive association between cognitive function and all-cause mortality. Compared to the participants with normal cognition and normal or excess weight, the participants who were both underweight and affected by CI had the highest risk of mortality after adjustment for potential confounders; this combined effect was more prominent among older adults aged <100 years and women than among centenarians or men. It should be noted that the association between BMI and mortality risk varied between older adults, depending on their cognitive function. To the best of our knowledge, this is the first study report the synergistic effects of BMI and cognitive function on the risk of all-cause mortality in older adults.

In this study, 26.09% of the participants were underweight, which is a much higher proportion than that previously reported in other large cohort studies (6, 7). Previous studies have reported on the independent association between BMI and all-cause mortality risk in older adults (2, 35, 36). A previous meta-analysis of studies focused on adults aged ≥65 years demonstrated a U-shaped association between BMI and mortality, whereby the lowest risk of death was detected among individuals with BMI between 24.0 kg/m^2^ and 30.9 kg/m^2^; in fact, no association was detected between high BMI (overweight) and increased risk of mortality (8). However, this meta-analysis included studies conducted in Europe, North America, Australia, and Canada. In China, studies on the population of adults aged ≥65 years (9, 37, 38) reported findings similar to those of our study, whereby high BMI (overweight/obesity) was not associated with increased risk of all-cause mortality. We are not certain that this finding supports the obesity paradox. Evidence has shown that the morbidity and mortality of various chronic diseases in obese people increase significantly. Compared with the normal weight, overweight/obese individuals tend to die earlier, thus survivor bias might exist in the older population in this study. In addition, the prevalence of obesity in the Chinese population is limited to the last 30 years, and it affects people with higher socioeconomic status more (9), who have easier access to better medical and health service. It may cover up the real association between high BMI and all-cause mortality. Furthermore, in contrast to high BMI, low BMI (underweight) had a significant association with all-cause mortality in this study. Evidence from a large cohort study has shown that weight loss might have a greater impact on the risk of mortality than weight gain in people aged >60 years (39). These findings may update policy development around health promotion and disease prevention among older adults in China.

Several previous studies have explored interaction effects of BMI and age (40), low-grade inflammation (41), and cardiovascular disease categories (42) on mortality risk in different populations. Our study expanded on this evidence, showing a significant interaction of BMI and cognitive function on all-cause mortality. Nevertheless, the present finding is inconsistent with that of a previous study, which did not find any interaction between BMI and the MMSE scores (43). However, this previous study included participants aged ≥70 years, residing in northern Italy; the study sample size was also relatively small.

The mechanism underlying the association between BMI, cognitive function, and all-cause mortality remains unclear. One potential explanation may be that low BMI (underweight) and CI may be surrogate indicators of underlying disease rather than direct contributors to mortality risk. In a study of 2,550 Chinese older adults aged ≥55 years, the prevalence of poor cognitive function was relatively high among people with low BMI and chronic comorbidity; meanwhile, low BMI was significantly associated with poor cognitive performance only in individuals with pre-existing chronic comorbidities (44). In addition, phenotypic and genetic associations have been reported between BMI and cognitive function, suggesting shared genetic basis and common biological pathways (22). The direction of phenotypic correlation indicated that lower BMI was associated with better cognitive function, which suggests that the relationship between BMI, cognitive function, and mortality risk is complex and likely mediated by several other factors.

A previous review has shown that the association between BMI and cognition, and mortality risk varies between life stages (45). Specifically, in the moderately old aged 75-84, high BMI (overweight and obesity) is associated with an increased risk of future dementia; however, in later life, this association changes, and higher BMI and better cognitive function begin to correspond to reduced mortality risk, in particular, when examining executive functions and language performance (46). This finding might help explain the discrepancy in findings between the centenarians included in the present study and the relatively younger adults; however, confirming this association requires further research.

Finally, higher body fat mass, which is closely related to higher BMI, has been reported to positively correlate with cognitive performance (47). Due to prominent exposure to endogenous estrogen, this association was more pronounced in older women than in men (48, 49). This finding might account for the differences in the present study findings between men and women.

The present findings are based on data from the long-term longitudinal study of community-dwelling general adult population in China. Low BMI (underweight), in particular, when concurrent with CI was significantly associated with increased all-cause mortality risk during the 7-year follow-up period. Based on this evidence, we suggest that appropriate anthropometric measurements and cognitive screening should be included in the health and care plan of older adults in China. Furthermore, in the present study, older adults with normal cognition experienced a stronger effect of low BMI (underweight) on the risk of mortality than their counterparts with CI. This finding suggests that maintaining optimal BMI is paramount also among older adults with normal cognitive function. Nevertheless, proactive assessment and improvement of cognitive function, as required, among individuals with low BMI (underweight) may help reduce their risk of premature mortality to a greater extent than a nutritional intervention alone.

The present study has several limitations. First, this study accounted for BMI only; no other anthropometric parameters such as body fat mass or distribution were considered. This limitation might have affected our ability to propose a mechanism underlying the presented associations, warranting further studies. Meanwhile, using a single indicator might have weakened our effect estimates because of the presence of regression dilution (50). However, excluding subjects with BMI >24 kg/m^2^ marginally affected the results, suggesting the validity of the presented estimates. Second, the present study relied on self-reported medical history, resulting in estimates of chronic diseases that were lower than those reported for the general older adult population in China. Therefore, the impact of chronic disease on the presented estimates (including confounding, interaction, and mediating effects) needs to be examined (44). Third, this study did not differentiate between causes of death; as a result, the presented mortality risks are composite estimates that preclude conclusions regarding specific cardiovascular or metabolic disease-associated risks that would be required to inform heath policies in these areas.

## Conclusions

In conclusion, the present study demonstrates that the general older adult population in China concurrently affected by low BMI (underweight) and CI is at the highest risk of premature all-cause mortality; within that group, women and older adults aged <100 years are at particularly high risk. Among the participants with normal cognition, the association between BMI and all-cause mortality was more prominent than among the participants with CI. These results suggest that cognitive screening is required for designing precise and effective interventions in this group. Further studies are required to validate these findings.

## Data Availability Statement

The datasets presented in this study can be found in online repositories. The names of the repository/repositories and accession number(s) can be found below: https://www.icpsr.umich.edu/icpsrweb/NACDA/series/487.

## Ethics Statement

The studies involving human participants were reviewed and approved by the Ethics Committee of Peking University and Duke University. The patients/participants provided their written informed consent to participate in this study. Written informed consent was obtained from the individual(s) for the publication of any potentially identifiable images or data included in this article.

## Author Contributions

The authors’ responsibilities were as follows—KH, WJ, SW, WC, SY, YS, JW, ML, and YH: designed the research. WC, YS, and JW: collected the data. KH, WJ, and SW: performed the statistical analysis. KH: wrote the paper. KH, WJ, and YH: had primary responsibility for the final content. All authors contributed to the article and approved the submitted version.

## Funding

The study was supported by Opening Foundation of State Key Laboratory of Kidney Diseases (KF-01-115), the National Natural Science Foundation of China (81773502, 81703285 and 81703308), Beijing Nova Program (Z181100006218085), the Medical Big Data Fund of Chinese PLA General Hospital (2018MBD-029) and the Opening Foundation of National Clinical Research Center of Geriatrics (NCRCG-PLAGH-2017017).

## Conflict of Interest

The authors declare that the research was conducted in the absence of any commercial or financial relationships that could be construed as a potential conflict of interest.
