# Supplementary material for: Synergistic Impact of Body Mass Index and Cognitive Function on All-Cause Mortality in Older Adults: A Nationwide Longitudinal Study
Source: Front Endocrinol (Lausanne). 2021 Jun 29;12:620261. doi: 10.3389/fendo.2021.620261 (PMC8276260; doi:10.3389/fendo.2021.620261)
Supplement: Supplementary file 1 [file DataSheet_1.docx]

Supplementary Material

# Supplementary Figures and Tables

## Supplementary Tables

### Table S1. Baseline characteristics of the participants by the results of follow-up

| **Characteristics** | **Total (n=9149)** | **Included in the analysis (n=8293)** | **Lost to follow-up (n=856)** | **P-value** |
| --- | --- | --- | --- | --- |
| Age (year) | 85.64 ± 11.15 | 85.53 ± 11.07 | 86.70 ± 11.86 | 0.003 |
| BMI (kg/m^2^) | 21.18 ± 4.30 | 21.20 ± 4.23 | 20.99 ± 4.91 | 0.167 |
| MMSE score | 22.69 ± 8.90 | 22.77 ± 8.82 | 21.97 ± 9.58 | 0.013 |
| Sex |  |  |  | 0.836 |
| Men | 4127 (45.11) | 3738 (45.07) | 389 (45.44) |  |
| Women | 5022 (54.89) | 4555 (54.93) | 467 (54.56) |  |
| Ethnicity |  |  |  | 0.913 |
| Han | 8654 (94.59) | 7845 (94.60) | 809 (94.51) |  |
| Others | 495 (5.41) | 448 (5.40) | 47 (5.49) |  |
| Residence |  |  |  | <0.001 |
| City | 1615 (17.65) | 1360 (16.40) | 255 (29.79) |  |
| Town | 2732 (29.86) | 2596 (31.30) | 136 (15.89) |  |
| Rural | 4802 (52.49) | 4337 (52.30) | 465 (54.32) |  |
| Years of education |  |  |  | 0.008 |
| <1 | 5327 (58.22) | 4865 (58.66) | 462 (53.97) |  |
| ≥1 | 3822 (41.78) | 3428 (41.34) | 394 (46.03) |  |
| Smoking |  |  |  | 0.288 |
| Current | 1667 (18.22) | 1528 (18.43) | 139 (16.24) |  |
| Former | 1468 (16.05) | 1327 (16.00) | 141 (16.47) |  |
| Never | 6014 (65.73) | 5438 (65.57) | 576 (67.29) |  |
| Alcohol drinking |  |  |  | <0.001 |
| Current | 1566 (17.12) | 1449 (17.47) | 117 (13.67) |  |
| Former | 1322 (14.45) | 1218 (14.69) | 104 (12.15) |  |
| Never | 6261 (68.43) | 5626 (67.84) | 635 (74.18) |  |
| Weekly exercise |  |  |  | 0.37 |
| Current | 3092 (33.80) | 2818 (33.98) | 274 (32.01) |  |
| Former | 1103 (12.06) | 990 (11.94) | 113 (13.20) |  |
| Never | 4954 (54.15) | 4485 (54.08) | 469 (54.79) |  |
| ADL impairment | 2300 (25.15) | 2028 (24.46) | 272 (31.81) | <0.001 |
| Spinal deformity | 3429 (37.48) | 3144 (37.91) | 285 (33.29) | 0.008 |
| Hypertension | 5181 (56.63) | 4718 (56.89) | 463 (54.09) | 0.115 |
| Diabetes | 376 (4.11) | 341 (4.11) | 35 (4.09) | 0.974 |
| Cardiovascular disease | 1126 (12.31) | 1011 (12.19) | 115 (13.43) | 0.292 |
| Stroke and cerebrovascular disease | 726 (7.94) | 642 (7.74) | 84 (9.81) | 0.033 |
| Respiratory disease | 1082 (11.83) | 979 (11.81) | 103 (12.03) | 0.844 |
| Cancer | 75 (0.82) | 65 (0.78) | 10 (1.17) | 0.235 |

**Data were reported as the mean (standard deviation) for continuous variables and number (%) for categorized variables.**

**Differences between two groups were evaluated by Student’s t-test or chi-square test.**

### Table S2. Hazard ratios for the combined associations of BMI and cognitive impairment with all-cause mortality stratified by age groups

|  | **HRs for all-cause mortality** | | | |
| --- | --- | --- | --- | --- |
|  | **65-79 years old(n=2801)** | **80-89 years old(n=2318)** | **90-99 years old(n=2047)** | **>100 years old(n=1127)** |
| Normal cognition |  |  |  |  |
| BMI≥18.5 | Ref. | Ref. | Ref. | Ref. |
| BMI<18.5 | 1.60 (1.27, 2.01) | 1.50 (1.29, 1.74) | 1.42 (1.23, 1.63) | 1.32 (1.07, 1.64) |
| CI |  |  |  |  |
| BMI≥18.5 | 2.19 (1.57, 3.07) | 1.99 (1.67, 2.38) | 1.68 (1.47, 1.93) | 1.57 (1.31, 1.89) |
| BMI<18.5 | 4.81 (3.03, 7.64) | 2.26 (1.80, 2.83) | 2.14 (1.83, 2.50) | 1.95 (1.61, 2.36) |
| P for interaction | 0.300 | 0.059 | 0.313 | 0.640 |

### Table S3. Sensitivity analyses for the combined associations of BMI and cognitive impairment with all-cause mortality

|  |  | **HR [95% CI] for all-cause mortality** | | | | |
| --- | --- | --- | --- | --- | --- | --- |
|  | **Additionally adjusting for** **medical service accessibility** | | **Additionally adjusting for sleep quality** | **Excluding the participants with spinal deformity** | **Excluding** **the participants whose BMI >** **24 kg/m^2^** | **Excluding the participants who died in the first year** |
| Normal cognition | | |  |  |  |  |
| BMI≥18.5 kg/m^2^ | Ref. | | Ref. | Ref. | Ref. | Ref. |
| BMI<18.5 kg/m^2^ | 1.63 (1.49, 1.79) | | 1.55 (1.42, 1.68) | 1.60 (1.42, 1.79) | 1.49 (1.36, 1.62) | 1.56 (1.43, 1.72) |
| CI |  | |  |  |  |  |
| BMI≥18.5 kg/m^2^ | 1.79 (1.63, 1.98) | | 1.79 (1.64, 1.96) | 1.92 (1.68, 2.19) | 1.79 (1.62, 1.98) | 1.80 (1.63, 1.99) |
| BMI<18.5 kg/m^2^ | 2.15 (1.92, 2.40) | | 2.18 (1.97, 2.41) | 2.39 (2.06, 2.77) | 2.12 (1.91, 2.36) | 1.95 (1.73, 2.20) |
| P for interaction | <0.001 | | <0.001 | 0.011 | 0.001 | <0.001 |

**HR: hazard ratio; BMI: body mass index; CI: cognitive impairment**

**Sensitivity analyses were based on the fully adjusted model for primary analysis.**

## Supplementary Figures

**

**

### Figure S1. Flowchart of the inclusion of participants.

MMSE, Mini-mental State Examination; BMI, body mass index

**
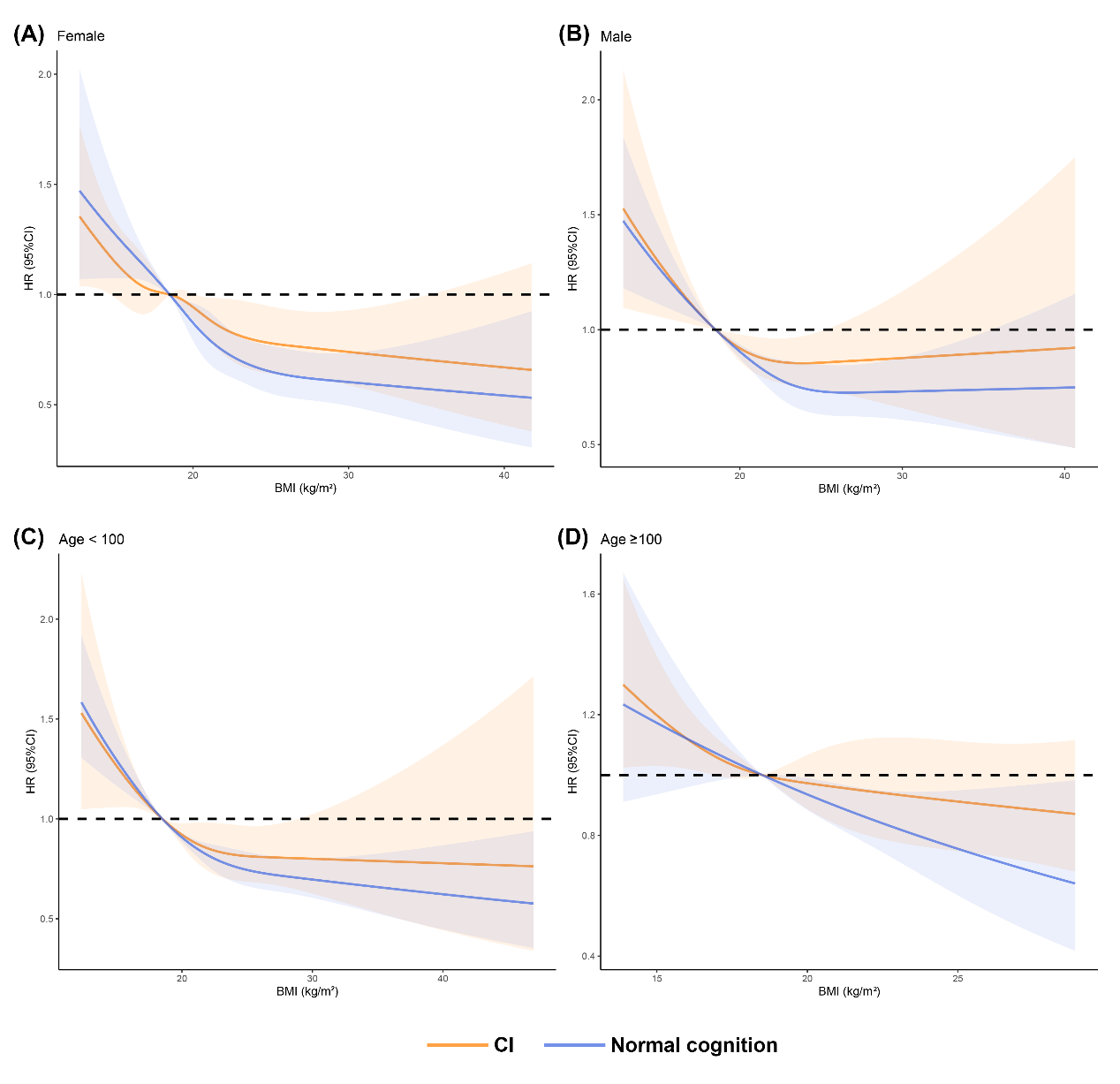
**

### Figure S2. Restricted cubic splines for the association between BMI and all-cause mortality by cognitive functions

(A) Association of BMI and all-cause mortality by cognitive functions in women

(B) Association of BMI and all-cause mortality by cognitive functions in men

(C) Association of BMI and all-cause mortality by cognitive functions in participants aged under 100

(D) Association of BMI and all-cause mortality by cognitive functions in participants aged 100 and above

All models were adjusted for age, sex, ethnicity, residence, education, smoking, alcohol drinking, weekly exercise, weekly exercise, spinal deformity, hypertension, diabetes mellitus, cardiovascular disease, stroke and cerebrovascular disease, respiratory disease and cancer
